# Review of methods to identify the critical job-tasks undertaken by the emergency services

**DOI:** 10.3233/WOR-192914

**Published:** 2019-08-17

**Authors:** Venturino R. Nevola, Maxwell D. Lowe, Colin A. Marston

**Affiliations:** Defence Science and Technology Laboratory (DSTL), Fareham, Hampshire, UK

**Keywords:** Physical Employment Standards, PES, occupation, performance, physical strain

## Abstract

**BACKGROUND:**

The roles of the emergency services are challenging and often physically demanding. *Readiness* to meet these challenges and demands is a fundamental requirement for staff to deliver their roles safely and effectively. Furthermore, employers are required by law to demonstrate every reasonable effort to protect their staff from undue risk of work-related injury. Implementing Physical Employment Standards (PES) enables employers to assign staff to roles for which they are physically-suited whilst contributing to such duty-of-care. However, for PES to be successful and legally-defendable, standards must reflect the demands of those job-tasks which are truly critical to the *readiness* of these services.

**OBJECTIVE:**

To determine whether a standardised approach to identifying critical job-tasks exists in the development of PES for the emergency services.

**METHODS:**

Studies which conducted analysis of job-tasks to develop PES within the emergency services were reviewed.

**RESULTS:**

Forty-two reported studies (i.e. records) met the inclusion criteria. Methods to determine job-tasks varied but were typically criteria-based incorporating one, or all, of 9 reported techniques. Methods were subjective and based upon reference to past or present job-task performance.

**CONCLUSION:**

Correctly determining critical job-tasks is essential for effective, legally-defendable PES. A standardised method to define job-tasks remains to be established.

## 1 Introduction

The concise Oxford English Dictionary defines an emergency as ‘*a serious, unexpected, and often dangerous situation requiring immediate action*’ (Tenth edition [2002], page 466). Alternative definitions describe incidents (events or situations) which may have already caused (or have a high probability of causing) an immediate threat to life, health/welfare, property, or the environment, including acts of war or terrorism [1]. The Civil Contingencies Act 2004 [1, 2] is an Act of Parliament in the United Kingdom (UK) that establishes a coherent framework for emergency planning and response. Other nations have similar policy and strategies (such as USA’s Homeland Security Act of 2002 [3, 4]). Common to all nations’ strategies is the nature of the emergency services as a collaboration of multiple agencies who are prepared to deliver the necessary immediate response to a wide range of incidents. The UK’s emergency response and recovery strategy [2] was used to define and categorise the roles emergency services are required to deliver. Category 1 *first responders* (i.e. organisations expected to arrive first on the scene of an incident) comprise: Police services; Fire and Rescue authorities; Paramedic / health agencies; Maritime and Coastguard Agency; local authorities; and Environment Agency. Category 2 responders concern the wider resilience community (including health professionals, transport providers, highways agency, telecommunications, Health and Safety Executive etc.). In the UK the military Armed Forces are mandated to support category 1 and 2 roles via the Military Aid to the Civil Authority (MACA) [5] as indeed the USA’s military personnel may similarly undertake the roles of first responders (akin to the UK’s category 1 response). For the purpose of this review the emergency services were classed as the category 1, or *first*, responders.

The roles of the emergency services are challenging and often physically demanding with a risk of injury [6]. Statistics for the UK Fire and Rescue Services reported annual (2018 [7]) response to 564,827 incidents (involving 167,150 fires) ranging from false alarms to major incidents. Manning strength at the start of the reporting year was 40,964 full time staff of which 33,049 were employed as firefighters [6]. By the end of the year 4,425 staff had left the service. Injuries sustained by firefighters in a similar period included 2,523 cases of which 1,071 injuries had been sustained during emergency operations.

In order to assure the necessary manning strength with which to deliver the operational capability there is a need to ensure that incumbents are able to undertake their roles safely and effectively. Furthermore, in order to satisfy employment law and to increase the size and scope of the population from which applicants may be recruited there is a need to diversify the workforce in terms of ethnicity, gender, age and ability. An example from the Fire and Rescue Service (England) reported [6] that female firefighters accounted for only 5.2% of incumbents and that only 3.9% of all firefighters represented ethnic minority groups. Methods to assess applicants and incumbents for their readiness to meet the physical demands of roles in the emergency services continue to evolve. Physical Employment Standards (PES) describe the level of physical performance that must be demonstrated during such assessments in order to meet the requirement for employment. In order to be defended in-Law, PES must reflect the actual demands of conducting job-tasks upon which operational success relies (i.e. the discrete actions or tasks which are undertaken as part of a job within the role of the emergency service).

The focus for this review were the methods used to identify those job-tasks which are critical to the emergency services, and upon which PES have been (or will be) established.

### 1.1 The purpose of Physical Employment Standards (PES): Why develop them?

Tipton et al. [8] state that the purpose of establishing a PES is to ensure that workers can complete the critical (in terms of performance and productivity) and generic (i.e. common or regular) tasks associated with their job without undue stress to themselves and to others working with them. Such purpose suggests that the physical demands of work may not be achieved by all people and that there is an associated risk of injury which may, within reason, be managed to its irreducible minimum. However, legislation mandating that employers enforce policies for the provision of health and safety at work (i.e. duty of care) and equal opportunities (without discrimination) have likely been the underlying drivers for PES since the 1970’s [9–11]. Recognising the risk of work-related injury and the need to maintain a safe, effective workforce of sufficient size, employers have increasingly invested in the development of PES as part of their selection process and to inform the training of recruits and incumbents. Efficiently placing people to the work for which they are best physically suited has been the premise of strategies to promote cost effectiveness in recruitment (as it is hoped that staff successfully complete their training at the first attempt, progress to deliver their roles without injury and remain in-service for the duration of their career). Increased legal scrutiny (since 2015) of the rationale for the exclusion (by many Armed Forces of various nations) of women from employment in the ground close combat roles emphasised the need for evidence-based PES.

### 1.2 The process for developing PES: How to develop them?

There are a number of well-established frameworks for the design and development of PES. The fundamental process for developing PES has been the topic of numerous technical events since the 1990’s [12–18]. Whilst researchers in Canada responded to legal scrutiny by establishing Bona Fide Occupational Requirements (BFOR) [12] other authors continued to report a similar process for developing physical fitness standards [19]. Whilst Gledhill et al. [12] had described the development of a BFOR in 12 stages and Taylor and Groeller [20] offered their generic planning model (incorporating trade- and task-analyses), the 6-stage process described by Tipton et al. [8] has been most often cited for PES:

Stage 1: Establish the critical job-tasks (i.e. job-analysis);
Stage 2: Determine the method of best practice for undertaking the critical job-tasks;
Stage 3: Agree the criteria-for, and acceptable minimum level of, job-task performance;
Stage 4: Determine the physical demands of the critical job-tasks;
Stage 5: Determine a reasonable maximum permissible relative workload; and
Stage 6: Production of a valid minimum occupational fitness standard.

Common to all approaches reported in the evidence-based literature is the need to correctly identify the critical job-tasks at the very start of the process. However, varied criteria have been reported for determining critical job-tasks and the methods used to acquire the evidence (i.e. qualitative and quantitative data).

### 1.3 The need to standardise the PES process

The precedent has been set within occupational research for use of the 6-stage process in developing PES [8, 21–24]. However, similar precedent is less evident with regard to the methods for conducting the initial stages of this process which are often contested in the Court of Law and must withstand legal scrutiny [24]. Reference to job analyses, task analyses, and trade analyses are commonplace for stage 1 in the process but they describe very different methods and data. Recognising the need for standardisation, researchers have proposed methods for each stage [22–24]. Payne and Harvey [23] provided their (often cited) comprehensive framework for the design of physical employment tests and standards, in which they discussed job-task analyses (considering component tasks and analysing the mode, frequency, duration, intensity and work: rest ratio). However, the description, by Tipton et al. [8], of job-task analysis as objectively subjective, highlighted the complexity of techniques that have been used to investigate this first stage in the PES process. Methods to select (and acquire data from) reliable, experienced, informed expert practitioners of the critical job-tasks have been suggested by leading researchers of PES [22, 24]. In 2016 an international delegation of these PES experts (representing 9 nations) formed a North Atlantic Treaty Organization (NATO) research task group [25] with the objective of designing an international standardisation agreement defining the methods to use when developing PES for combat roles.

### 1.4 Job-task analysis

A job-task analysis (sometimes known as a job analysis, task analysis or work analysis) has been defined [21, 26] as the process for establishing an accurate accounting of the tasks or activities that take place in a job. No published guidelines exist to instruct researchers on how to conduct job-task analyses [21, 27] and there is no single correct method that fits all requirements. Hardison et al. [21] recommended that the choice of method may necessarily vary according to the intended purpose of the analysis (e.g. to develop PES or to review doctrine). They [21] added that the centrality of a job-task analysis in defending the use of a selection system or PES could not be overstated. There was a fundamental requirement to correctly identify and characterise the job-tasks which were evidently critical to the success of the emergency service. Furthermore, the job-task analysis was considered to be fundamental to ensuring that the standards for an occupation were valid predictors of critical job requirements (such assurance was considered essential in order to withstand legal scrutiny [28]). Landy and Vasey [29] reported that plaintiffs would typically assert that there was a fatal flaw in the job analysis techniques, analyses, results, or inferences. Thus, the choice of method used to define the content of the job would be vital in addressing some of the criticisms that might be raised. Larsen and Aisbett [27] asserted that given the importance of reporting accurate and legally defensible job-task analyses it was imperative to critically evaluate methods and to provide a best-practice approach for future research.

Hardison et al. [21] reviewed several methods used to develop PES for the Armed Forces. The techniques that were cited when conducting job analyses included; [a] analysis of documentation such as job descriptions, training manuals and task inventories (i.e. the employer’s list of tasks that were in-scope for specific jobs); [b] review of relevant scientific literature and professional competencies; [c] conduct of site visits to observe, and to interview, incumbents performing their jobs; [d] interviews with instructors and job-supervisors; [e] judgement panels comprising Subject Matter Experts (SME); and [f] on-line staff surveys. Zumbo [30] suggested criteria to apply when selecting SMEs as suitably qualified and experienced professionals. Use of technology (where participants rated the performance of scenario-based tasks that were observed via recorded imagery) enabled Siddall et al. [31] to acquire real-time data from anonymized e-voting systems within their expert judgement panels. Methods were cited which acquired both qualitative and quantitative data [8] and included ratings of task importance, difficulty, intensity, duration and frequency.

### 1.5 Objective of this study

To identify the methods by which critical job-tasks have been determined when developing PES within the emergency services and to assess whether a standardised approach may exist.

## 2 Method

A review of the evidence-based literature was conducted using six information systems: (a) Web of Science v5.30; (b) National Center for Biotechnology Information’s PubMed; (c) Google Scholar; (d) United Kingdom’s (UK) defence research database known as ATHENA; (e) Defense Technical Information Center (DTIC^®^ which is an open-access repository managed by the United States of America’s Department of Defense); and (f) Scientific publications available from Australia’s Defence Science and Technology (DST) Group website. Searches were limited to documents that had been reported between 01 January 1976 and 31 July 2018. Search terms (i.e. keywords) used in this study were identified following a preliminary search of the literature (using PubMed). Assurance that the keywords were appropriate and inclusive was assumed following a further search of the literature which successfully acquired several publications (of varying citation) that were known to meet the study’s inclusion criteria. The keywords used in this study were: Physical Employment Standard (PES); job-task; job analysis; review; emergency services; method; first responder; physical performance; emergency services; fire; ambulance; paramedic; police; government; military and Bona Fide Occupational Requirement (BFOR). To find additional studies, the reference lists of the articles obtained were also investigated. Only information that was reported by each study was included in this review. No additional information was sought from the authors of the reported studies. An initial search was conducted to determine whether such a review had already been reported in the open literature.

### 2.1 Eligibility for the review

Titles and abstracts of the acquired studies were assessed for their eligibility against the following criteria:

a. Qualification for review required the following conditions to be met (i.e. *inclusion* criteria):

• Reported within the search dates (i.e. between 01 January 1976 and 31 July 2018);
• Written in English;
• Studies reporting methods to identify essential, or critical, physically-demanding job-tasks within the emergency services;
• Novel studies at their first reporting with the objective to inform the development of PES at the time of reporting or in the near future;
• Evidence of peer-review;
• Context relevant to PES (keyword search).

b. Records were *excluded* if they were:

• Classified documents which the owner would not authorise for release;
• Instruction manuals or reference documents;
• Not applicable to the emergency services;
• Unable to identify one or more critical job-tasks;
• Lacking in sufficient detail to describe the techniques used in the method;
• Reviews of, or proposals for, ‘best-practice’ when developing PES which did not report previously unreported data from a novel study;
• A duplicate report of a study which had appeared in another publication;
• Incomplete or draft manuscripts and documents which had not undergone peer-review.

The above criteria formed a checklist which was used by two of the authors of this review to independently assess the studies for their eligibility. Only those studies which were assessed by both authors as eligible were included in the review.

### 2.2 Processing the reported methods

Details of the methods reported were entered into a Microsoft Excel workbook. Each reported method was assigned to one of the following {1} to {9} categories:

{1} Surveys
i.e. questionnaires, focus groups, interviews, workshops etc;
{2} Facilitated expert judgement panels
e.g. military judgement panels;
{3} Review of policy and doctrine
including training manuals;
{4} Historical analysis
i.e. Use of data which reported the course of action, and performance, of the emergency services when managing past incidents. This included incidents reports, after-action-reviews and recorded lessons;
{5} Case law
Considering litigation and any legal precedent concerning the legitimacy of defined job-tasks as essential, or critical, to the role of the emergency service (and underpinning a legally-defendable PES);
{6} Observation and analysis of real-life work and/or training exercises
Use of research staff to observe and record details of tasks undertaken by trained incumbents conducting their role in a real, or realistic (i.e. training), scenario;
{7} Job descriptions and performance appraisals
i.e. Definitions used by the emergency service to describe specific jobs and the criteria that incumbent peers (and managers) apply to assess the quality of staff performance
{8} Consultation with Subject Matter Experts (SME) and stakeholder panels
e.g. Access to appointed liaison officers and stakeholder working groups that are empowered to provide evidence-based advice to the study team (distinct from {1} or {2} by the assignment of a specific SME advisor to the project and/or by direct involvement with the employer’s working group or committee)
{9} Other reported methods
e.g. Mathematical modelling, role-play and research staff conducting the training courses intended to prepare personnel for a role within the emergency service

A process for assessing (e.g. scoring) the quality of implementation for each reported method was not conducted.

### 2.3 Analyses

The methods that were reported by each of the studies which successfully met the eligibility criteria were recorded by their category ({1} to {9}) and quantity (i.e. the number of methods used from 1 [minimum] to 9 [maximum]). The frequency with which each individual method was reported (between studies) was calculated as a proportion (%) of the total number of eligible studies in the review. The Microsoft Excel data analysis histogram tool was used to determine the frequency (calculated as a proportion [% ] of the eligible studies) with which *multiple methods* had been reported. The statistical mode was calculated for the number of methods reported by the studies (data were not adjusted for heterogeneity).

## 3 Results

### 3.1 The literature search

**Fig.1:**
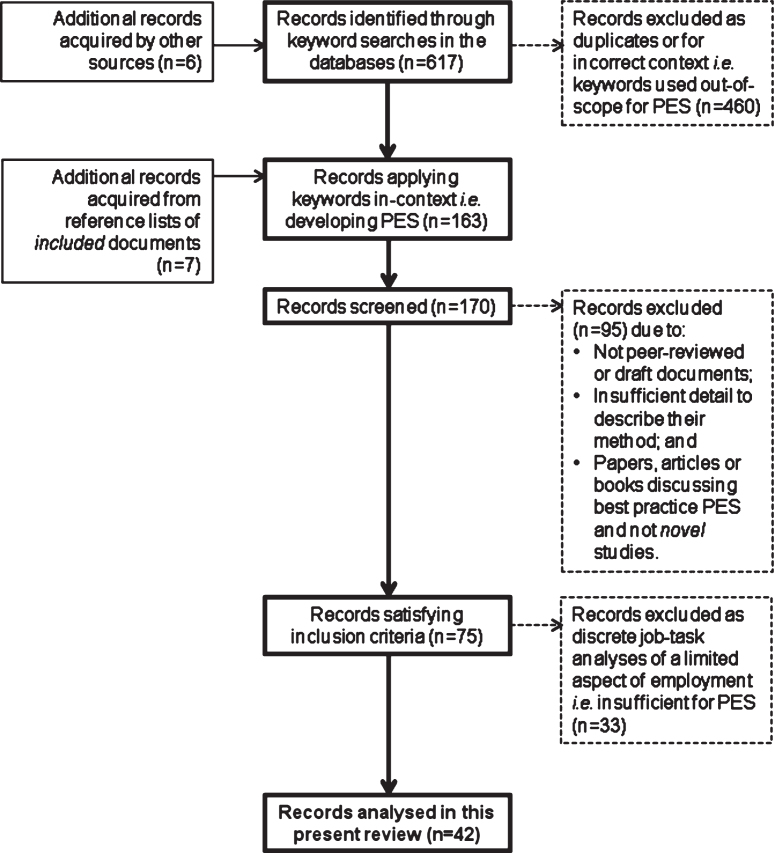
Results of each stage of the process in assessing eligibility for inclusion in this present review.

Use of the keywords in the initial search identified 617 records. No previously reported review was identified which sufficiently met the objective of this present study. A further 6 records were acquired from other (manual) sources which included proceedings from scientific conferences. Hence, a total of 623 records were assessed for their eligibility against the inclusion/exclusion criteria. Figure 1 presents the outcome of each stage of the assessment process which resulted in the independent assessments by 2 of the authors of this present study concurring that 42 of the records qualified for this review.

### 3.2 Findings from the studies that were reviewed

**Fig.2:**
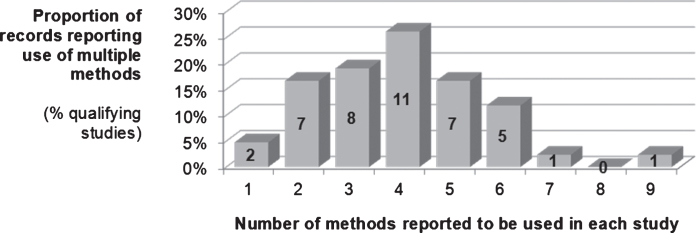
Use of methods to identify critical job-tasks in the emergency services (as reported by the [*n* = 42] studies in this review).

**Fig.3:**
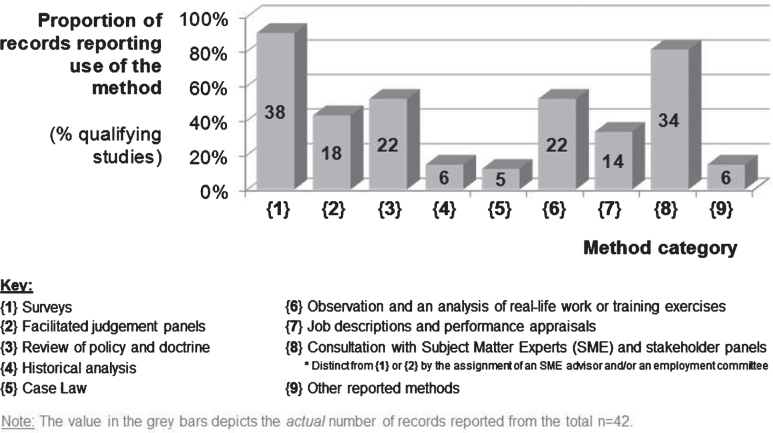
Number (and proportion) of the 42 studies reporting use of multiple methods to identify critical job tasks in the emergency services.

The number and type of methods used by each of the 42 records included in this review have been described in Table 1. The value for the mode number of methods or techniques reported by a record was 4 (methods reported ranged from 1 [least] to 9 [most]). Twenty-five records reported using 4 or more method categories (Fig. 2), whilst 2 of the remaining 17 records relied exclusively upon the use of surveys (i.e. applied only 1 method). Within the 42 studies that were reviewed the use of survey methods were evident in 38 (90%) cases whilst consultation with Subject Matter Experts (SME) and stakeholder panels (empowered to make decisions regarding the emergency service), i.e. {8}, was cited in 34 (81%) cases (Fig. 3).

**Table 1:** Methods (reported by 42 studies) to identify critical job-tasks in the development of

Qualifying study	Participant population	EMERGENCY SERVICE	Methods to identify job-tasks used in PES (see * key)									Additional information	Considered *future* job-tasks? (Y/N)	Nation
			1	2	3	4	5	6	7	8	9 (Other)			
Blacker et al. [35]	Ground Close Combat	MILITARY (COMBAT)	x	x	x	x	x	x	x	x	Review of mission tasks lists.		**Y**	UK
Milligan [43]	Maritime Coast Guard Agency	(NAVY)	x	x	x		x	x	x	x			N	UK
Nevola et al. [44]	BAA fire fighters	FIRE	x		x			x	x	x	Developed a list of core operational tasks.	Incumbent and instructor-informed job performance criteria.	**Y**	UK
Nevola et al. [45]	Police (Home Office)	POLICE	x		x		x	x	x	x		Identified core role related patrol tasks. Used Police competency framework.	**Y**	UK
Nevola et al. [46]	Royal Air Force	AIR	x	x	x			x	x	x			N	UK
Gruse et al. [47]	Explosive Ordnance Disposal (EOD)	SPECIALIST		x	x	x		x	x	x		Access to task inventories.	N	USA
Gledhill and Jamnik [48]	Firefighters	FIRE	x	x	x			x	x	x			N	CAN
Myers et al. [49]	All Arms Army	ARMY	x	x	x				x	x			N	UK
Taylor et al. [50]	Urban Firefighters	FIRE	x	x	x				x	x			N	AUS
Foulis et al. [51]	Army Combat Arms	MILITARY (COMBAT)	x		x	x			x	x		Military Occupational Specialisations (MOS) lists.	N	USA
Kilding and Fordy [52]	Army	ARMY	x	x	x				x	x			N	NZ
Siddall et al. [31]	Firefighters and commanders	FIRE	x	x			x	x		x			N	UK
Reilly [53]	Royal National Lifeboat Institute crew	NAVY	x		x			x	x	x			N	UK
Vine et al. [54]	Royal Marine Commando	MILITARY (COMBAT)	x	x	x			x		x			N	UK
Allen et al. [55]	Armed Forces	MILITARY (COMBAT SUPPORT)	x		x			x		x		Panel of extramural expert advisors in PES.	N	CAN
Blacker et al. [56]	Fire and Rescue	FIRE	x	x	x					x		Consultation with policy makers.	N	UK
Deakin et al. [57]	Armed Forces	MILITARY (COMBAT SUPPORT)		x		x				x	Applied previous definitions of critical tasks (used in PES).	Developed common emergency tasks.	N	CAN
Doyle et al. [58]	Defence Force	MILITARY (COMBAT SUPPORT)	x			x		x		x			N	AUS
Gumieniak [59]	Wildfire fighters	FIRE	x				x			x	Developed scenarios.		N	CAN
Marshall-Mies et al. [60]	Department of Defense adjudicators	SPECIALIST	x		x				x	x		Developed task list (database).	N	USA
Milligan et al. [61]	Offshore Wind Industry	INDUSTRY	x		x			x		x			N	UK
Prusaczyk et al. [62]	Sea, Air, Land (SEAL) personnel	NAVY	x	x		x				x			N	USA
Rayson et al. [34]	Army	ARMY	x		x			x		x			N	UK
Silk et al. [63]	Police	SPECIALIST	x	x	x					x		Applied delphi method.	N	AUS
Sothmann et al. [42]	Firefighters	FIRE	x	x				x		x		Task inventory included cognitive requirements.	N	USA
Fischer et al. [64]	Paramedics	PARAMEDIC	x					x		x			N	CAN
Middleton et al. [65]	Royal Australian Navy	NAVY	x	x						x			N	AUS
Redmond et al. [66]	Infantry Indirect Fire Battery	ARMY	x		x					x		Military Occupational Specialisations (MOS) lists.	**Y**	USA
Silk and Billing [67]	Defence Force Dismounted Assault Task	MILITARY (COMBAT)			x				x	x			N	AUS
Robson et al. [68]	Tactical Air Control Party and Air Liaison Officers	SPECIALIST	x	x						x			**Y**	USA
Jamnik et al. [69]	Correction Officers	POLICE	x	x						x		Applied delphi method to responses structured into emergency scenarios.	N	CAN
Doolittle et al. [70]	Line Workers	SPECIALIST	x					x		x			N	CAN
Osborn [71]	Police	POLICE	x					x			Researchers conducted the incumbent training course to familiarise with job-tasks.		N	USA
House et al. [72]	Royal Navy Divers	NAVY	x							x			N	UK
Stein et al. [73]	Special Weapons And Tactics (SWAT)	SPECIALIST	x							x		SME moderator of information.	N	USA
Stuart-Hill et al. [74]	Marine Search and Rescue	NAVY	x					x					N	CAN
Stevenson et al. [75]	Armed Forces	MILITARY (COMBAT SUPPORT)			x			x					N	CAN
Smith [76]	Royal Navy	NAVY	x					x					N	UK
Anderson et al. [77]	Police	POLICE	x					x					N	CAN
Reilly et al. [78]	Royal National Lifeboat Institute beach lifeguards	NAVY	x								Mathematical modelling.	Theoretical analysis of time requirements.	N	UK
Anderson et al. [79]	Police	POLICE	x										N	CAN
Brown and Fallowfield [80]	Royal Navy	NAVY	x									Analysis of a generic task list.	N	UK

* Key (methods): **{1}** Surveys (questionnaires, focus groups, interviews, workshops); **{2}** Facilitated expert judgement panels (including military judgement panels); **{3}** Review of policy and doctrine (including training manuals); **{4}** Historical analysis of past reported emergencies; **{5}** Case law; **{6}** Observation and analysis of real-life work and/or training exercises; **{7}** Job descriptions and performance appraisals; **{8}** Consultation with Subject Matter Experts (SME) and stakeholder panels; and **{9}** Other reported methods.

In 22 (52%) cases the studies described observing incumbents conducting their service roles, i.e. {6}, (either during operational duty or scheduled training exercises) as well as seeking assurance by cross-reference to policy and doctrine, i.e. {3} (established tactics, techniques and procedures for use in both training and emergency scenarios).

The miscellaneous methods which were categorised within {9} included: (a) research staff undertaking the training of the emergency service (to gain understanding and practical experience of the tasks and their associated physical demands); (b) use of mathematical modelling techniques; (c) development of operational scenarios in which the critical job-tasks were conducted; (d) development, or review, of operational task lists; and (e) reference to critical job-tasks which had been defined by previous research. Thirteen (31%) records cited the use of task lists developed by, or on behalf of, the emergency service (e.g. Military Occupational Specialisations [MOS], and Mission Essential Task Lists [METL]). Only 5 (12%) records sought information to describe how job-tasks may be conducted in future scenarios (obtained by method category {1}, {8} or {9}), or acquired guidance from SMEs regarding strategic plans for the immediate future of the emergency service (e.g. the planned introduction of new equipment and operating procedures). Records (*n* = 37 [88%]) tended to limit methods to acquiring evidence from past or present operations and doctrine.

## 4 Discussion

The results support the position that no standardised method or criteria have been reported to determine critical job-tasks for the purpose of developing PES in the emergency services. However, these results were limited to inclusion of only those documents baring the security classification which permitted access by the aforementioned methods. Authors of the records included in this review described constraints in the availability of (and access to) information managed within (or on behalf of) the employing emergency service which limited their methods. Military and security services (particularly maritime) accounted for the overriding majority of records which were eligible for this review whilst certain elements of the emergency services (e.g. environment agency etc.) were notable by the lack of published information to describe their job-tasks. Method categories {4} and {5} (i.e. historical analyses and case law, respectively) received fewest citations within the reported methods. Whilst completed legal cases challenging PES within each emergency service may have been comparatively few it was surprising that analyses of empirical data from operations were rarely considered (despite surveys typically requesting incumbents to provide subjective ratings of their operational performance) [81].

### 4.1 Standardising the identification of critical job-tasks: Factors to consider

Dukalskis and Beadle [32] suggested that there were many commonly used methods for systematically obtaining details regarding a job, task or role (i.e. job analysis [33]). However, the choice of method varied and depended upon the type of information that was hoped to be gained and the purpose of the investigation. It was evident that the variance between services regarding the availability of appropriately managed information and data was an important factor in the cases that were reviewed (e.g. MOS; competency frameworks; training manuals; incident reports and investigations; incident lessons; performance databases; continuous professional development records etc.). Hence, any future intent to standardise the methods for determining critical job-tasks [25] (and their performance criteria) must encourage employers to maintain robust and accessible information management systems. Operational data are needed to understand the determinants of success and failure when describing job-task performance.

It is likely that PES will be subjected to further legal challenge in the future. Subsequent reviews of this nature should consider the methods that were used to establish critical job-tasks in cases where PES was successfully defended. Where such methods are found to be consistently successful in their legal defence, they should be incorporated as best practice when developing PES.

Establishment of an international standard (for developing PES) will facilitate data sharing and help to determine reliable success criteria when setting standards or cut-off scores in performance assessments. However, a paradox may exist where the intent to reflect the reality of conduct on operations is in conflict with the method of best practice as advocated in doctrine and underpinning PES. An example of a physically demanding job-task has included the replacement of a wheel on a truck [34]. Although doctrine may describe procedures for loosening the wheel nuts using upper body muscle strength, in practice applying body weight to the wrench (by standing on it) may reflect the reality of common practice on operations and result in a lesser requirement for upper body muscle strength. Implications for strict adherence to doctrine (in such cases) when setting PES need to be understood and explained to the employer.

### 4.2 Implications of strategic plans on the priority and performance of future job-tasks

It can take several years to develop PES [24, 34, 35]. Strategic review of the roles of the emergency services (including procurement of new equipment, infrastructure, protective clothing and operating procedures) is typically conducted within a similar timescale [36–38]. Therefore, criteria used to develop PES must remain relevant to the operational roles for which they are expected to represent else they risk being out-of-date as soon as they are implemented [82]. When designing a BFOR, Gledhill et al. [12] stated that the job-task analysis needed to understand the evolution of the job both past and future as well as considering the changing nature of the job (including legitimate variations in how the job-tasks may be achieved). Authors of guidance concerning the process for developing BFOR and PES suggest the need to consider future tasks but they do not offer any specific methods for achieving this. Only 5 of the 42 records included in this review considered the implications of future developments in their critical job-tasks. However, employers undertaking strategic review of the roles in the emergency services typically apply techniques such as Wargaming [39] and red teaming to assess resilience and to investigate future ways of operating. Wargaming roles [39] and scenarios [83] planned for the future workforce (an established scenario-based model where the outcome and sequence of events affect, and are affected by, the decisions made by SME players) was not evident in any study in this review. Wargaming facilitates realistic role-play for specific scenarios [83] which may allow incumbents to repeatedly practice common incidents as well as those emergencies which may occur only rarely in a typical career. Existing guidance for conducting job-task analyses when developing PES [40] does not consider the techniques (such as Wargaming) that are increasingly used by these employers.

Reference to the emergency services describes the collaboration of multiple agencies responding to the same incident. However, PES may differ between services despite the similarity in the emergency job-tasks they conduct. Although some differences may be reasonably defended, there remains a risk of legal challenge which would be wise to anticipate and address [84]. Furthermore, emergency services rarely, if ever, deploy individuals to manage an incident. However, PES are applied to individual performances. Consideration should be given to determinants for effective team working as well as individual competence.

### 4.3 Potential opportunities provided by emerging technology

PES that are valid are those which correctly distinguish between people who are likely to be able to perform the requirements of the job from those who are not. Hardison et al. [21] suggest that without careful implementation and ongoing monitoring and updating, even well designed standards will fail to screen individuals appropriately if the testing is done improperly or as occupational tasks and equipment change over time. Emerging technology is increasingly enabling personnel to be monitored in ways that had not previously been possible. Wearable sensors and stand-off sensing continue to develop rapidly and may soon enable the concept of rolling validation (i.e. on-going evaluation of the physical demands of job-tasks) to be achieved [85]. GPS-based techniques for conducting notational analysis of individuals operating in large complex teams is becoming increasingly available to the emergency services. Process may endure but the methods must progress with such advances in technology in order to benefit from greater ability to monitor true work performance and to improve the sensitivity and specificity of PES.

### 4.4 Employment standards an holistic approach

In 1999 Dukalskis and Beadle [32] developed the FLAG system (a system for conducting a job analysis based upon Fleishman’s algorithm [41]). Their system recognised that jobs are not purely physical in nature but require timely and effective coordination of various competencies (e.g. cognitive and physical). Future standards may need to consider an holistic approach [86] to assessing job-task performance rather than discriminating individual competencies in isolation. Sothmann et al. [42] included cognitive tasks within their job-task inventory as did Padula et al. [86].

### 4.5 Recommendations

The results suggest that there is a need for the emergency services to:

1 [a] Maintain robust and accessible information with which to describe their critical job-tasks;
2 [b] Adopt a consistent approach (i.e. international standardisation) to the development of performance standards which accurately reflect the combination of competencies required to safely and successfully conduct critical job-tasks;
3 [c] Implement employment standards which incorporate the requirements of job-tasks that have been assessed to be critical to both present and likely future operational scenarios (e.g. informed by techniques such as wargaming).

Research to inform techniques which develop and assure employment standards should consider emerging advances in practical, pervasive monitoring technologies (including smart technologies/environments and wearable systems). There is also a need to develop reliable methods to assess the contribution (and performance) of individuals whilst they work in teams during real-time operations. Methods must be applicable to all emergency services and must be readily integrated within their operational systems (without unduly adding to the demands of conducting the job-tasks). Data from real-time operations (undertaken by the emergency services) remain an enduring and critical requirement in the assurance of evidence-based employment standards.

## 5 Conclusion

Correctly determining critical job-tasks is essential for legally-defendable PES. No consistent method was evident to determine critical job-tasks for those studies included in this review. Criteria used to establish critical job-tasks were based upon subjective judgement of past and present performance. A standardised method to define job-tasks when developing PES remains to be established and should include techniques used to inform future roles and strategic force development (e.g. wargaming).

## Conflict of interest

None to report.
